# Effectiveness of Case-Based Learning Versus Lecture-Based Learning in Enhancing Critical Thinking and Retention in Pharmacology: A Mixed-Methods Cross-Sectional Study From Mauritius

**DOI:** 10.7759/cureus.109374

**Published:** 2026-05-21

**Authors:** Saanvee Makarand Sapte, Krishna Dodia, Rudraksh Sharma, Navita Jatain, Indrajit Banerjee

**Affiliations:** 1 Pharmacology, Sir Seewoosagur Ramgoolam Medical College, Belle Rive, MUS

**Keywords:** case-based learning, case-based teaching, clinical case, indian ocean islands, lecture-based learning, medical school students, pharmacology and therapeutics, pharmacology learning, traditional didactic lecture, undergraduate medical student

## Abstract

Introduction: Pharmacology is a well-established branch of medicine taught in the preclinical years, encompassing the study of drugs, their mechanisms of action, dosing, adverse effects, and therapeutic applications. The primary objective of this mixed-methods cross-sectional study was to evaluate the comparative effectiveness of case-based learning (CBL) versus lecture-based learning (LBL) in pharmacology at Sir Seewoosagur Ramgoolam Medical College, Mauritius.

Materials and methods: A mixed-methods cross-sectional study was conducted at Sir Seewoosagur Ramgoolam Medical College in Mauritius from September to December 2025. Quantitative data were processed and analyzed using SPSS Statistics version 31.0 (IBM Corp., Armonk, NY, USA). Qualitative data were analyzed using NVivo 15 software (QSR International, Melbourne, Australia) through inductive thematic analysis.

Results: A total of 318 of 336 students participated in the quantitative component of the study, yielding a response rate of 94.64%. The mean age of participants was 22.71 ± 1.56 years. Overall, 129 (78.2%) female and 134 (87.6%) male students preferred CBL, whereas 36 (21.8%) female and 19 (12.4%) male students preferred LBL, citing that it was easier to follow without prior preparation (p = 0.027). Similarly, 137 (83.0%) female and 139 (90.8%) male students preferred CBL, while 28 (17.0%) female and 14 (9.2%) male students preferred LBL for reducing confusion or misunderstanding about the topic (p = 0.040). Clinical-phase students were more likely to prefer CBL for future sessions compared with preclinical students (adjusted OR 3.720, 95% CI 1.095-12.637; p = 0.035). Male students were more likely than female students to report that CBL was easier to follow without prior preparation (adjusted OR 2.003, 95% CI 1.024-4.020; p = 0.040) and that it reduced confusion or misunderstanding (adjusted OR 1.968, 95% CI 1.074-3.608; p = 0.042). Thematic analysis of qualitative data generated six key themes: cognitive engagement, application, recall, metacognition, interaction, and perceptions of learning format.

Conclusions: This study demonstrates that students in both preclinical and clinical semesters, and across genders, generally preferred CBL over traditional LBL. Participants reported that CBL was easier to follow without prior preparation and helped reduce confusion or misunderstanding in pharmacology learning.

## Introduction

Pharmacology is a well-established branch of medicine taught during the preclinical years, focusing on drugs, their mechanisms of action, dosages, adverse effects, and therapeutic uses. In medical education, pharmacology plays a crucial role, bridging students’ understanding of pharmacological principles and their ability to apply them in clinical practice. This knowledge is essential for future doctors to prescribe medications safely and effectively. At Sir Seewoosagur Ramgoolam Medical College, affiliated with the University of Mauritius, pharmacology is taught to second-year undergraduate students through lectures, practical sessions, and tutorials [1].

Pharmacology is primarily delivered through lecture-based learning (LBL), also known as didactic lectures, a teacher-centered method of instruction. This approach enables the efficient delivery of a large volume of structured information, providing students with an organized framework for study. However, previous evidence suggests that LBL alone is insufficient to develop essential clinical competencies in medical students, as it promotes passive learning, is often monotonous, and offers limited opportunities for active engagement, discussion, problem-solving, and independent inquiry. Prior research has demonstrated that active learning strategies, such as problem-based approaches, outperform traditional lecture-based methods in promoting student learning [2].

Several innovative teaching and learning strategies have been introduced and evaluated, including problem-solving sessions and interactive seminars, with evidence generally favoring these newer approaches. Pharmacology, in particular, requires conceptual understanding, strong recall, effective memorization, and critical thinking, skills that may be better developed through case-based learning (CBL). CBL is a student-centered approach that emphasizes active learning and encourages learners to apply theoretical knowledge to real-world clinical scenarios, thereby promoting higher-order cognitive skills [2]. This model fosters collaborative engagement between faculty and students, shifting the focus toward learner-centered education. It not only bridges the gap between theoretical knowledge and clinical application but also enhances student motivation and knowledge retention through active participation in the learning process [3].

The need for CBL in pharmacology is increasingly recognized worldwide. Early clinical exposure and case-based illustrations help students connect foundational biomedical sciences with real patient scenarios, thereby improving knowledge retention. This approach enhances students’ ability to analyze clinical information and apply pharmacological knowledge in patient management through active participation [4]. Furthermore, CBL promotes critical thinking by encouraging students to evaluate clinical data, consider differential explanations, and make evidence-based decisions. These processes contribute to the development of metacognitive skills. Additionally, CBL supports self-directed learning by encouraging students to independently seek, evaluate, and apply knowledge in clinical reasoning and decision-making [4].

Problem statement

Most existing comparative studies between CBL and LBL rely primarily on quantitative methods, such as pre- and post-knowledge assessments or Likert-scale perception surveys, and do not adequately capture the subjective and experiential aspects of student learning [5,6]. In Mauritius, there is a paucity of data comparing CBL and LBL in pharmacology education. This study was designed to address this gap. By adopting a mixed-methods design that combines quantitative evaluation with qualitative thematic analysis of semi-structured interviews, the study provides a more comprehensive and triangulated understanding of the educational phenomenon, capturing both learning outcomes and students’ lived learning experiences.

Objective of the study

The primary objective of this mixed-methods cross-sectional study was to evaluate the comparative effectiveness of CBL versus LBL in pharmacology across four cognitive domains: knowledge retention, critical thinking, metacognition, and self-directed learning at Sir Seewoosagur Ramgoolam Medical College, Mauritius.

This article was previously presented as a pilot project with a smaller sample size at the Health Horizon 2025: 5th International Symposium on Medical Sciences on 06 September 2025 in Mauritius.

## Materials and methods

Research design

A mixed-methods cross-sectional study was conducted at Sir Seewoosagur Ramgoolam Medical College in Mauritius from 16 September to 15 December 2025. A total of 318 of 336 students participated in the quantitative component of the study, yielding a response rate of 94.64%. For the qualitative component, data were collected from 14 students (seven males and seven females), with two students selected from each semester. Participants were anonymized and labeled P01-P14. The study was reported in accordance with the Strengthening the Reporting of Observational Studies in Epidemiology (STROBE) guidelines [7].

Questionnaire design, validity, and reliability

Following an extensive literature review, a questionnaire was developed in alignment with the study objectives. The instrument was partially adapted from a previously validated questionnaire by Garg and Bhanwra [8] and modified for contextual relevance to Mauritius for the quantitative component. A panel of four experts assessed content, construct, and criterion validity. The average congruence percentage was 90%, indicating strong content validity.

Cronbach’s alpha was used to assess internal consistency reliability. The values were 0.833 for the CBL experience domain and 0.862 for the LBL experience domain, indicating good reliability and acceptable internal consistency.

The questionnaire comprised five sections: (a) demographic characteristics, (b) experience with CBL, (c) experience with LBL, (d) preference for CBL versus LBL in the quantitative domain, and (e) qualitative open-ended questions (Appendix). A 5-point Likert scale was used to assess agreement (1 = strongly disagree, 2 = disagree, 3 = neutral, 4 = agree, 5 = strongly agree).

Data collection

Quantitative data were collected using Google Forms (Google LLC, Mountain View, CA, USA). Qualitative data were obtained through semi-structured interviews using a standardized set of open-ended questions developed by the researchers under the supervision of a senior author (Professor). Participants were informed of the study objectives and procedures prior to data collection. They were also informed that interviews would be audio-recorded and transcribed, with participants' anonymity maintained, and that all data would be used exclusively for research purposes. Written informed consent was obtained from all participants.

Sampling technique and sample size calculation

The required sample size was calculated using the formula:

$z^2 p(1-p)/d^2$ 

where n = sample size, z = standard normal variate corresponding to a 95% confidence interval, p = expected population proportion, and d = margin of error.

Assuming a 95% confidence level (Z = 1.96), a margin of error of 5% (d = 0.05), and a conservative estimated proportion of 0.75, the sample size was calculated as:

$n=(1.96)^2&sdot;0.75(1-0.75)/(0.05)^2 =288$

Thus, a minimum sample size of 288 participants was required. To account for potential nonresponse and improve statistical power, the sample size was increased by 10%, resulting in a final target of 316 participants.

For the qualitative component, data collection followed the principles outlined by Glaser and Strauss [9], and interviews continued until data saturation was reached, defined as the point at which no new codes or themes emerged.

Inclusion and exclusion criteria

Students in semesters 4-10 at Sir Seewoosagur Ramgoolam Medical College, Mauritius, who were currently studying or had previously studied pharmacology were included in the study. Participation was voluntary. Students from semesters 1 to 3 were excluded because they had not yet been exposed to pharmacology.

Outcome and explanatory variables

The primary outcome variables were exposure to CBL and LBL, as well as associated student preferences. Explanatory variables included age, gender (male and female), nationality (India, South Africa, and Mauritius), semester (4-10), and phase of study (preclinical and clinical).

Ethical consideration

The study adhered to the principles of the Declaration of Helsinki for research involving human participants, ensuring confidentiality, anonymity, and voluntary participation. Ethical approval was obtained from the Institutional Research and Ethics Committee of Sir Seewoosagur Ramgoolam Medical College, Mauritius, on 15 September 2025 (Approval Code: SSRMC/IERB/2025/005).

Data management and statistical analysis

Quantitative Domain

Data were analyzed using SPSS Statistics version 31.0 (IBM Corp. Released 2025. IBM SPSS Statistics for Windows, Version 31.0. Armonk, NY: IBM Corp.). Descriptive statistics, including percentages and 95% confidence intervals, were computed. Associations between variables were assessed using the chi-square test and logistic regression analysis. A p-value < 0.05 was considered statistically significant.

Qualitative Domain

Qualitative data were analyzed using Braun and Clarke’s six-step thematic analysis framework [10]. NVivo 15 software (QSR International, Melbourne, Australia) was used to support inductive thematic analysis and facilitate data coding and theme development.

## Results

Quantitative domain

A total of 318 of 336 students participated in the quantitative component of the study, yielding a response rate of 94.64%. The mean age of participants was 22.71 ± 1.56 years. Of the respondents, 165 (51.9%) were female and 153 (48.1%) were male. Regarding the phase of study, 115 (36.2%) students were in the preclinical phase, while 203 (63.8%) were in the clinical phase. In terms of nationality, 163 (51.3%) were Indian, followed by 100 (31.4%) Mauritian students (Table 1).

**Table 1:** Demographic details Data are represented as N (%). CI: confidence interval

Variable	Category	N (%)	95% CI
Gender	Male	153 (48.1)	42.6, 53.6
	Female	165 (51.9)	46.4, 57.4
Country	India	163 (51.3)	45.8, 56.8
	Mauritius	100 (31.4)	26.3, 36.5
	South Africa	55 (17.3)	13.1, 21.5
Phase of study	Preclinical	115 (36.2)	30.9, 41.5
	Clinical	203 (63.8)	58.5, 69.1

Tables 2-3 show that participants reported higher mean scores for CBL compared with LBL. For the first statement, the mean score for CBL was 3.87, higher than that for LBL (2.65). The standard deviation for CBL (±0.84) was lower than that for LBL (±1.03), indicating that responses for CBL were more consistent and demonstrated less variability.

**Table 2:** Mean score and SD for CBL Data are represented as mean ± SD. CBL: case-based learning, SD: standard deviation

Statements for CBL	N	Mean	SD
I could remember details from the classes even after a few days without revisiting the material.	318	3.87	±0.84
The learning format helped me evaluate multiple possible answers before reaching a conclusion.	318	3.96	±0.69
This format improved my ability to apply pharmacological concepts in real-life clinical cases.	318	4.05	±0.73
This learning format increased my motivation to explore pharmacology topics independently.	318	3.95	±0.78
This learning format helped me monitor and adjust my learning strategy while studying.	318	3.99	±0.77
I could retrieve previously learned information during related discussions or assignments.	318	4.04	±0.76
After classes, I feel more confident applying pharmacological knowledge effectively during examinations.	318	4.01	±0.77

**Table 3:** Mean score and SD for LBL Data are represented as mean ± SD. LBL: lecture-based learning, SD: standard deviation

Statements for LBL	N	Mean	SD
I could remember details from the classes even after a few days without revisiting the material.	318	2.65	±1.03
The learning format helped me evaluate multiple possible answers before reaching a conclusion.	318	2.67	±1.06
This format improved my ability to apply pharmacological concepts in real-life clinical cases.	318	2.74	±1.09
This learning format increased my motivation to explore pharmacology topics independently.	318	2.66	±1.07
This learning format helped me monitor and adjust my learning strategy while studying.	318	2.74	±1.09
I could retrieve previously learned information during related discussions or assignments.	318	2.69	±1.15
After classes, I feel more confident applying pharmacological knowledge effectively during examinations.	318	2.77	±1.14

Table 4 shows that the mean composite score for CBL (27.9 ± 4.2) was higher than for LBL (18.9 ± 6.7), indicating that CBL was perceived more favorably by participants and was associated with greater engagement and active knowledge retention. In contrast, the lower composite scores for LBL suggest that it was perceived as less effective in promoting engagement and active retention.

**Table 4:** Composite scores of total mean and total SD Data are represented as mean ± SD. CBL: case-based learning, LBL: lecture-based learning, SD: standard deviation

Variable	Composite mean score	SD	N
CBL	27.9	±4.2	318
LBL	18.9	±6.7	318

**Figure 1:**
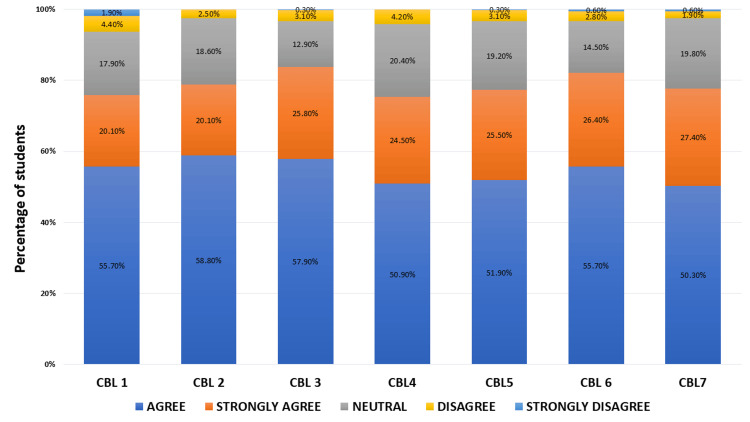
CBL (Likert scale) The data are represented as percentages (%). CBL 1-7 (Appendix). CBL: case-based learning (%)

Figure 1 shows the distribution of student responses based on Likert-scale analysis for CBL. Overall, responses indicated a strong positive perception, with most students selecting “agree” or “strongly agree” across all domains (Q1-Q7). The proportion of “agree” responses ranged from approximately 50.3% to 58.8%, while “strongly agree” responses ranged from 20.1% to 27.4%, resulting in a combined positive response rate of 71% to 85%.

Negative responses were minimal, with “disagree” ranging from approximately 1.9% to 4.4% and “strongly disagree” from 0.3% to 1.9%, indicating very low levels of dissatisfaction. Neutral responses ranged from approximately 12.9% to 20.4%, particularly in Questions 4 and 7, suggesting some variability in perceptions.

Overall, the response pattern indicates that CBL was highly favored by students, with strong agreement, minimal disagreement, and greater perceived effectiveness, engagement, and acceptance than traditional LBL.

**Figure 2:**
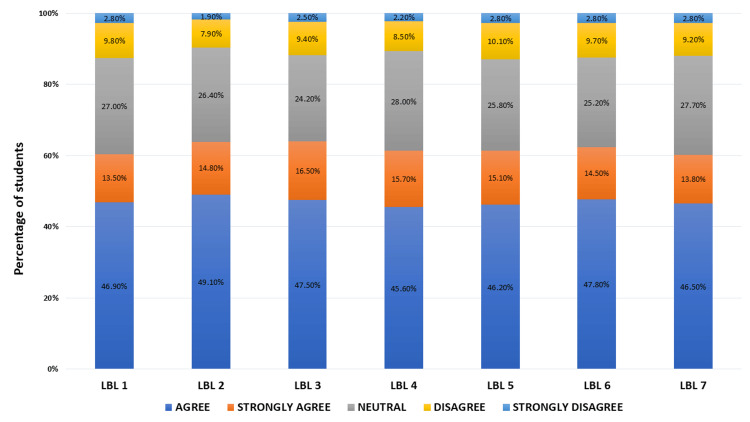
LBL (Likert scale) The data are represented as percentages (%). LBL 1-7 (Appendix). LBL: lecture-based learning

Figure 2 shows the distribution of student responses based on the Likert scale for LBL. Overall, the responses demonstrate noticeable variability across all domains. Most students selected “agree,” with proportions ranging from approximately 45.6% to 49.1%, followed by “strongly agree,” ranging from 13.5% to 16.5%. However, these positive responses were consistently lower than those reported for CBL.

A relatively high proportion of students selected “neutral” (approximately 24.2% to 28.0%), which was higher than in CBL, indicating weaker preference and lower confidence in LBL. Additionally, more negative responses were observed for LBL, with “disagree” ranging from 7.9% to 10.1% and “strongly disagree” from 1.9% to 2.8%. These values were higher than those seen for CBL, suggesting lower levels of engagement and satisfaction.

Taken together, while LBL elicited generally positive responses, the lower agreement levels, higher neutral responses, and increased disagreement indicate that it was less preferred and perceived as less effective than CBL in promoting active engagement and strong student approval.

Table 5 presents a cross-tabulation of students’ preferences for CBL or LBL by gender and phase of study. Regarding the perception that the learning format was easier to follow without prior preparation, 129 (78.2%) female and 134 (87.6%) male students preferred CBL, whereas 36 (21.8%) female and 19 (12.4%) male students preferred LBL. This difference was statistically significant (p = 0.027).

**Table 5:** Cross-tabulation between gender and phase of study with preferred learning format (CBL versus LBL) ^X ^p > 0.05: statistically insignificant, ^** ^p < 0.05: statistically significant Chi-square test (χ²) is used to calculate the p-values. CBL: case-based learning, LBL: lecture-based learning

Items related to preference for CBL or LBL	Variable	Category	CBL n (%)	LBL n (%)	Chi-square value	p-value
Which learning format did you find more effective for understanding the topic?	Gender	Female	155 (93.9%)	10 (6.1%)	χ² = 0.103	0.748^X^
		Male	145 (94.8%)	8 (5.2%)		
	Phase of study	Preclinical	109 (94.8%)	6 (5.2%)	χ² = 0.132	0.936^X^
		Clinical	191 (94.4%)	12 (5.6%)		
Which format would you prefer for future sessions?	Gender	Female	157 (95.2%)	8 (4.8%)	χ² = 1.091	0.296^X^
		Male	149 (97.4%)	4 (2.6%)		
	Phase of study	Preclinical	107 (93%)	8 (7%)	χ² = 5.037	0.081^X^
		Clinical	199 (98.1%)	4 (1.9%)		
Which format was easier to follow without prior preparation?	Gender	Female	129 (78.2%)	36 (21.8%)	χ² = 4.904	0.027**
		Male	134 (87.6%)	19 (12.4%)		
	Phase of study	Preclinical	93 (80.9%)	22 (19.1%)	χ² = 0.610	0.737^X^
		Clinical	170 (83.7%)	33 (16.3%)		
Which format helped reduce confusion or misunderstanding about the topic?	Gender	Female	137 (83.0%)	28 (17.0%)	χ² = 4.234	0.040**
		Male	139 (90.8%)	14 (9.2%)		
	Phase of study	Preclinical	97 (84.3%)	18 (15.7%)	χ² = 1.062	0.588^X^
		Clinical	179 (88.1%)	24 (11.9%)		
Which learning format kept you more engaged during the classes?	Gender	Female	162 (98.2%)	3 (1.8%)	χ² = 1.277	0.258^X^
		Male	147 (96.1%)	6 (3.9%)		
	Phase of study	Preclinical	110 (95.7%)	5 (4.3%)	χ² = 1.523	0.467^X^
		Clinical	199 (98.1%)	4 (1.9%)		

Similarly, regarding the perception that the learning format reduced confusion or misunderstanding about the topic, 137 (83.0%) female and 139 (90.8%) male students preferred CBL, while 28 (17.0%) female and 14 (9.2%) male students preferred LBL. This association was also statistically significant (p = 0.040).

Table 6 presents the logistic regression analysis examining the association between preference for CBL or LBL, gender, and phase of study. Clinical-phase students were more likely to prefer CBL for future sessions compared with preclinical students (adjusted OR 3.720, 95% CI 1.095-12.637; p = 0.035).

Similarly, male students were more likely than female students to report that CBL was easier to follow without prior preparation (adjusted OR 2.003, 95% CI 1.024-4.020; p = 0.029) and that it reduced confusion or misunderstanding about the topic (adjusted OR 1.968, 95% CI 1.074-3.608; p = 0.042).

**Table 6:** Logistic regression table X p > 0.05: statistically insignificant, ** p < 0.05: statistically significant Logistic regression analysis was performed to calculate the adjusted OR. CBL: case-based learning, LBL: lecture-based learning, OR: odds ratio, CI: confidence interval

Items related to preference for CBL or LBL	Variable	Category	Adjusted OR	95% CI	P-value
Which learning format did you find more effective for understanding the topic?	Gender	Female	1	0.449-3.044	0.759^x^
		Male	1.169		
	Phase of study	Clinical	0.876	0.320-2.400	0.797^x^
		Preclinical	1		
Which format would you prefer for future sessions?	Gender	Female	1	0.560-6.435	0.304^x^
		Male	1.898		
	Phase of study	Clinical	3.720	1.095-12.637	0.035**
		Preclinical	1		
Which format was easier to follow without prior preparation?	Gender	Female	1	1.074-3.608	0.029**
		Male	1.968		
	Phase of study	Clinical	1.219	0.672-2.211	0.515^x^
		Preclinical	1		
Which format helped reduce confusion or misunderstanding about the topic?	Gender	Female	1	1.024-4.020	0.042**
		Male	2.003		
	Phase of study	Clinical	1.384	0.716-2.676	0.334^x^
		Preclinical	1		
Which learning format kept you more engaged during the classes?	Gender	Female	1	0.111-1.847	0.270^x^
		Male	0.454		
	Phase of study	Clinical	2.261	0.595-8.595	0.231^x^
		Preclinical	1		

**Figure 3:**
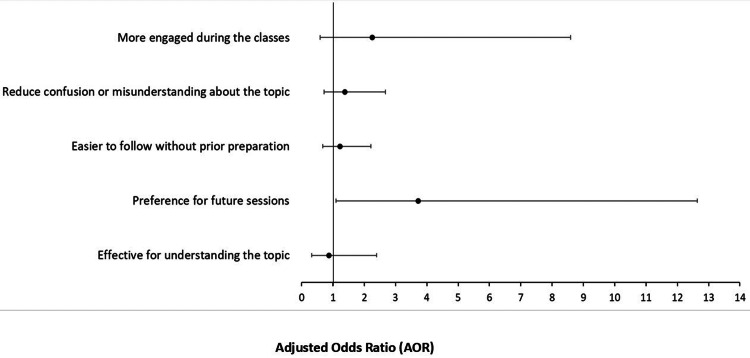
Forest plot of phase-wise analysis comparing preclinical and clinical groups Preclinical phase (reference/1).

Figure 3 presents the forest plot comparing clinical and preclinical students. Clinical-phase students were significantly more likely to prefer the CBL format for future sessions compared with preclinical students (adjusted OR 3.720, 95% CI 1.095-12.637; p = 0.035).

In contrast, no statistically significant differences were observed for other outcomes, including understanding of the topic, ease of following without prior preparation, reduction in confusion or misunderstanding, and engagement during classes, as the confidence intervals crossed the no-effect line and the p-values were greater than 0.05.

**Figure 4:**
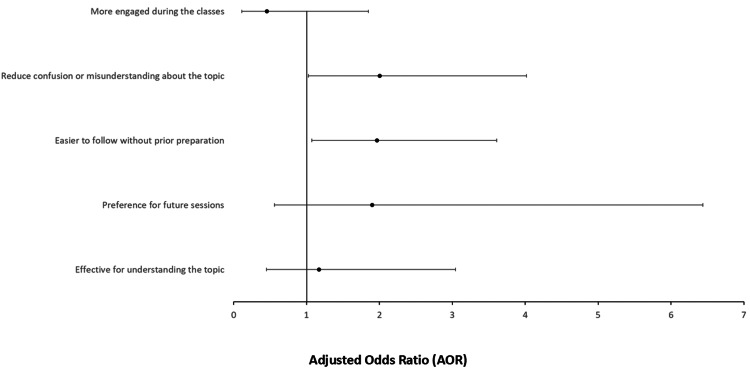
Forest plot of gender-wise analysis for preference toward CBL Female (reference/1).

Figure 4 presents the gender-wise forest plot analysis. Male students were significantly more likely than female students to report that the CBL format was easier to follow without prior preparation (adjusted OR 2.003, 95% CI 1.024-4.020; p = 0.042) and that it helped reduce confusion or misunderstanding about the topic (adjusted OR 1.968, 95% CI 1.074-3.608; p = 0.042).

However, no statistically significant gender differences were observed in understanding of the topic, preference for future sessions, or engagement during classes, as the corresponding confidence intervals crossed the no-effect line.

Qualitative domain

Qualitative data were collected from 14 students, comprising seven males and seven females, with two students selected from each semester. Participants were from semesters four to ten and were anonymized as P01-P14. The interviews were transcribed, reviewed, and thematically analyzed. Themes were developed through coding into nodes and subcodes, as presented in Table 7. The identified themes included cognitive engagement, application, recall, metacognition, interaction, and perception of learning format.

**Table 7:** Thematic analysis and codes

Themes	Nodes/codes	Subcodes
Cognitive engagement	Deep processing	Conceptual understanding
		Analytical thinking
	Motivation	Interest
		Curiosity
	Sustained attention	Focus
		Alertness
	Passive learning	Monotony
		Low stimulation
	Reduced attention	Distraction
		Fatigue
Application	Clinical reasoning	Diagnosis
		Treatment decision
	Knowledge transfer	Real-life application
		Case linkage
	Management planning	Drug selection
		Dosage decisions
	Clinical context	Patient scenarios
		Symptom interpretation
	Limited application	Lack of contextualization
		Symptom confusion
Recall	Long-term retention	Concept retention
		Clinical recall
	Contextual recall	Case-triggered recall
		Scenario association
	Visual retention	Image association
		Patient linkage
	Forgetting	Poor recall
		Exam difficulty
	Short-term retention	Temporary memory
		Surface learning
Metacognition	Confidence	Answering confidence
		Clinical confidence
	Learning Strategies	Mnemonics
		Note-making
	Self-reflection	Error identification
		Diagnostic reasoning
	Self-directed learning	Independent research
		Self-application
	Memorization strategy	Repetition
		Rote learning
Interaction	Peer learning	Group discussion
		Collaborative learning
	Active participation	Questioning
		Answering
	Facilitator interaction	Teacher guidance
		Feedback
	Engagement environment	Two-way communication
		Interactive setting
Perception of format	CBL preference	Practical learning
		Engagement
	LBL limitation	Boredom
		Confusion
	Blended approach	Integration
		Complementary learning
	Flexibility	Open-ended reasoning
		Exploration
	Structure	Organized format
		Systematic teaching
	Content coverage	Complete syllabus
		Theoretical breadth
	Visual enhancement	Images
		Case visuals

Most students perceived CBL as an effective and preferred learning method, as shown in Table 8. In contrast, traditional LBL was described as monotonous, with limited student engagement and poorer knowledge recall.

**Table 8:** Codes with their descriptions and corresponding narratives

Codes	Description	Narrative
Deep processing	Higher-order thinking and understanding	“helps you think more… deeper thinking” (P02)
Motivation	Increased interest and engagement	“CBL was comparatively… more interesting and motivated us” (P05)
Sustained attention	Ability to stay focused	“you stay awake and focus more when it's a case” (P03)
Passive learning	Lack of engagement in lectures	“monotonous… repetitive” (P06)
Reduced attention	Loss of concentration	“I’m already half asleep” (P12)
Clinical reasoning	Ability to interpret cases and decide diagnosis/treatment	“helps you look at the whole scenario… before you think of medications” (P10)
Knowledge transfer	Applying theory to real-life or clinical settings	“when you see the case in hospital… you think about the drugs” (P08)
Management planning	Deciding treatment strategies and drug use	“we know that these drugs will treat these ones” (P09)
Clinical context	Understanding concepts within patient scenarios	“you remember discussing the case” (P03)
Limited application	Difficulty applying lecture knowledge	“we get lost… everything says nausea and vomiting” (P014)
Long-term retention	Ability to retain knowledge over time	“you remember CBL” (P11)
Contextual recall	Memory triggered by cases	“you remember discussing the case” (P013)
Visual retention	Memory linked to visual or patient association	“I associate the drug with that person” (P03)
Forgetting	Poor recall of lecture content	“you don’t quite recall” (P07)
Short-term retention	Temporary memory retention	short term retention… traditional” (P02)
Confidence	Confidence in applying knowledge	“you confident in giving an answer” (P04)
Learning strategies	Use of techniques like mnemonics	“make small mnemonics” (P08)
Self-reflection	Evaluating reasoning and decisions	“you start thinking like, why it’s not this disease, and finding answers to it” (P01)
Self-directed learning	Independent study beyond class	“you do a bit of research and study by yourself” (P06)
Memorization strategy	Rote memorization approach	“traditional is more like repetition” (P05)
Peer learning	Learning through group interaction	“group discussions with my friends helped me” (P12)
Active participation	Engagement in discussion	“everyone else putting in their input during class” (P06)
Facilitator interaction	Interaction with teachers	“interacting with the teachers is something we learned” (P08)
Engagement environment	Interactive classroom setting	“case-based interactive learning observed” (P05)
CBL preference	Preference for CBL	“CBL easier to follow” (P10)
LBL limitation	Drawbacks of lectures	“it gets you confused… bored” (P09)
Blended approach	Preference for combining methods	“combine both CBL and traditional is something we need” (P03)
Flexibility	Open-ended exploration	“it was more flexible compared to lectures (P05)
Structure	Organized lecture format	“different subtitles and format were very structured and easy to follow” (P02)
Content coverage	Broad theoretical coverage	“uses, adverse effects, everything was covered easily” (P12)
Visual enhancement	Importance of visual aids	“image based learning… should be motivated” (P11)

The codes generated from the qualitative analysis included deep processing, motivation, sustained attention, passive learning, reduced attention, clinical reasoning, knowledge transfer, management planning, clinical context, limited application, long-term retention, contextual recall, visual retention, forgetting, short-term retention, confidence, learning strategies, self-reflection, self-directed learning, memorization strategies, peer learning, active participation, facilitator interaction, engagement environment, CBL preference, LBL limitation, blended approach, flexibility, structure, content coverage, and visual enhancement (Table 8).

## Discussion

Quantitative domaini

This mixed-methods cross-sectional study evaluated the effectiveness of CBL compared with LBL among undergraduate medical students. The findings consistently demonstrate a clear preference for CBL across all assessed domains, including retention, critical thinking, memory, clinical application, metacognition, and self-directed learning. These results support the ongoing shift toward active, student-centered learning strategies in medical education.

One of the most notable findings is the difference in composite scores between CBL and LBL. Students reported higher CBL scores in retention, the ability to evaluate multiple possible answers, the application of pharmacological concepts to clinical scenarios, and confidence in examinations. In contrast, LBL consistently demonstrated lower mean scores, suggesting that traditional lectures may be less effective in promoting active and meaningful learning. The higher consistency in CBL responses, reflected by lower standard deviations, further indicates a more uniform positive perception among students [11].

These findings are consistent with previous literature. A systematic review by Thistlethwaite et al. concluded that CBL is an effective teaching strategy in health professions education, particularly in enhancing clinical reasoning and bridging the gap between theory and practice [12]. Similarly, Srinivasan et al. reported that students exposed to CBL demonstrated improved clinical knowledge and more positive attitudes toward learning than those in traditional teaching methods [13]. Collectively, these findings reinforce the value of CBL as an effective instructional approach in pharmacology education.

In the present study, students rated CBL more favorably for critical thinking and knowledge retention, particularly for recalling information without revision and for evaluating multiple possible solutions before reaching a conclusion. These results are consistent with Kamal et al., who demonstrated that CBL significantly improves critical thinking and clinical competence among medical students [14]. Furthermore, a systematic review by Varma et al., which included 22 comparative studies, also found that CBL significantly enhances critical thinking skills [15]. Unlike many of these studies, which relied primarily on objective test scores, the present study used a validated Likert-scale instrument, allowing for a more comprehensive assessment of students’ perceived learning experiences.

Student engagement emerged as another key finding, with more than half of participants reporting that CBL enhanced engagement during pharmacology sessions and facilitated active participation in discussions. This aligns with findings by Kaur et al., who reported that CBL improves student engagement and attendance in pharmacology compared with LBL [6]. In the present study, although attendance data were not directly assessed, the strong preference for CBL serves as an indirect indicator of increased motivation and engagement. Importantly, the inclusion of both preclinical and clinical students across multiple semesters suggests that the benefits of CBL are consistent across different stages of medical training.

CBL was also strongly associated with self-directed learning and metacognitive development. Students reported improved ability to plan, monitor, and regulate their learning, which are essential components of effective clinical practice [16]. These findings are consistent with Demirören et al., who reported that active learning strategies such as CBL significantly improve metacognitive skills and academic performance compared with traditional lecture-based methods [17].

Gender differences were also observed in this study. While both male and female students generally preferred CBL, significant differences were identified in two domains: ease of following the format without prior preparation and reduction of confusion or misunderstanding. These findings suggest that gender may influence how students perceive structured versus active learning environments.

Similarly, the phase of study was significantly associated with preference for CBL in terms of ease of following the format. Clinical-phase students demonstrated a stronger preference for CBL, likely due to their greater exposure to patient care and ability to relate theoretical knowledge to clinical scenarios. This is consistent with Thomas et al., who reported that perceived clinical relevance significantly enhances knowledge retention in CBL-based curricula.

However, the overall phase of study did not independently predict learning format preference across all domains. This suggests that while clinical exposure influences certain aspects of learning perception, it does not fully determine overall preference, indicating that learning needs may be more complex and multifactorial.

Compared with previous studies, most of which relied on pre-test/post-test designs and limited quantitative metrics, the present study provides a broader perspective by incorporating both objective and subjective dimensions of learning. Importantly, it captures students’ lived learning experiences, motivations, and perceptions, factors that are often underexplored in traditional educational research.

This study addresses this gap by employing a mixed-methods design in a large, multicultural cohort spanning seven semesters and both preclinical and clinical phases. To the best of our knowledge, this is one of the few studies in the region to simultaneously evaluate CBL versus LBL across four cognitive domains: knowledge retention, critical thinking, metacognition, and self-directed learning. Additionally, the use of logistic regression to examine the influence of gender and phase of study adds analytical depth not commonly seen in similar studies.

Qualitative domain

The qualitative analysis revealed six major themes: cognitive engagement, application, recall, metacognition, interaction, and perception of learning format. Students reported that CBL promoted deep processing, sustained attention, and active participation. In contrast, LBL was frequently described as monotonous and passive, and as associated with reduced attention.

CBL was also perceived to enhance clinical reasoning, knowledge transfer, and management planning through exposure to clinical scenarios. It effectively bridged the gap between theoretical knowledge and clinical practice, a critical requirement in medical education. Conversely, LBL was viewed as having limited practical application, contributing to weaker retention and a disconnect between theory and clinical relevance.

Students further reported that CBL supported long-term retention and contextual recall, whereas LBL was associated with short-term retention and increased forgetting. CBL also contributed to the development of metacognitive skills and self-directed learning, with students describing increased confidence, self-reflection, and independent learning strategies that supported clinical decision-making. In contrast, LBL was more commonly associated with rote memorization.

Additionally, CBL fostered collaborative learning, peer interaction, and active discussion, enhancing the overall learning environment. While LBL was appreciated for its structured format and comprehensive content delivery, it was perceived as lacking interaction and engagement.

Overall, the preference for CBL observed in this study is consistent with findings from Garg et al. and Kaur et al., who also reported improved engagement, satisfaction, and critical thinking with CBL in pharmacology education [4,8].

The inclusion of qualitative data strengthens this study by providing insight into students’ lived experiences, which are often not captured in quantitative assessments alone. The integration of survey data, interviews, and mixed cohorts enhances the robustness of the findings. Despite the strong preference for CBL, students acknowledged the value of LBL for providing structured, comprehensive content delivery. This supports the potential benefit of a blended learning approach that integrates the strengths of both methods.

Strengths of the study

A key strength of this study is its mixed-methods design, which integrates quantitative survey data with qualitative interview findings. This approach allowed for triangulation of results and provided deeper insight into student perceptions and learning experiences. Additionally, to the best of our knowledge, this is the first study conducted in Mauritius to compare CBL and LBL across four cognitive domains: knowledge retention, critical thinking, metacognition, and self-directed learning.

Limitations of the study

This study was conducted in a single institution, which may limit the generalizability of the findings to other medical schools or educational settings. Additionally, the effectiveness of a blended approach combining CBL and LBL was not evaluated.

Future research recommendations

Future studies should adopt a multicentric design with larger sample sizes to improve generalizability. Further research is also recommended to evaluate blended learning approaches integrating CBL and LBL. Expanding the scope to include multiple disciplines across both preclinical and clinical medical education would also provide a more comprehensive understanding of teaching effectiveness.

## Conclusions

This study demonstrates that CBL yields higher mean scores and lower variability than traditional LBL, indicating greater perceived effectiveness. Participants from different semesters and both genders consistently preferred CBL, while LBL was less favored overall. Students reported that CBL was easier to follow without prior preparation and reduced confusion or misunderstanding in pharmacology topics. Additionally, clinical-phase students were more likely than preclinical students to prefer CBL for future learning sessions.

## Appendices

**Table 9:** Questionnaire comparing CBL and LBL in pharmacology CBL: case-based learning, LBL: lecture-based learning

Sr. no.	Section A: Demographics							
1.	Age							
2.	Gender	Female	Male					
3.	Nationality	India	South Africa	Mauritius				
4.	Semester	IV	V	VI	VII	VIII	IX	X
5.	Phase of study	Preclinical (semester IV-VI)	Clinical (semester VII-X)					
Section B: CBL								
CBL1.	I could remember details from the classes even after a few days without revisiting the material.	Strongly disagree	Disagree	Neutral	Agree	Strongly agree		
CBL2.	The learning format helped me evaluate multiple possible answers before reaching a conclusion.	Strongly disagree	Disagree	Neutral	Agree	Strongly agree		
CBL3.	This format improved my ability to apply pharmacological concepts in real-life clinical cases.	Strongly disagree	Disagree	Neutral	Agree	Strongly agree		
CBL 4.	This learning format increased my motivation to explore pharmacology topics independently.	Strongly disagree	Disagree	Neutral	Agree	Strongly agree		
CBL5.	This learning format helped me monitor and adjust my study strategy.	Strongly disagree	Disagree	Neutral	Agree	Strongly agree		
CBL 6.	I could retrieve previously learned information during related discussions or assignments.	Strongly disagree	Disagree	Neutral	Agree	Strongly agree		
CBL 7.	After classes, I feel more confident in applying pharmacological knowledge effectively in the examination.	Strongly disagree	Disagree	Neutral	Agree	Strongly agree		
Section C: LBL								
LBL1.	I could remember details from the classes even after a few days without revisiting the material.	Strongly disagree	Disagree	Neutral	Agree	Strongly agree		
LBL2.	The learning format helped me evaluate multiple possible answers before reaching a conclusion.	Strongly disagree	Disagree	Neutral	Agree	Strongly agree		
LBL 3.	This format improved my ability to apply pharmacological concepts in real-life clinical cases.	Strongly disagree	Disagree	Neutral	Agree	Strongly agree		
LBL 4.	This learning format increased my motivation to explore pharmacology topics independently.	Strongly disagree	Disagree	Neutral	Agree	Strongly agree		
LBL 5.	This learning format helped me monitor and adjust my learning strategy while studying.	Strongly disagree	Disagree	Neutral	Agree	Strongly agree		
LBL 6.	I could retrieve previously learned information during related discussions or assignments.	Strongly disagree	Disagree	Neutral	Agree	Strongly agree		
LBL 7.	After classes, I feel more confident in applying pharmacological knowledge effectively in examinations.	Strongly disagree	Disagree	Neutral	Agree	Strongly agree		
Section D: Preference								
1.	Which learning format did you find more effective for understanding the topic?	CBL	LBL					
2.	Which format would you prefer for future sessions?	CBL	LBL					
3.	Which format was easier to follow without prior preparation?	CBL	LBL					
4.	Which format helped reduce confusion or misunderstanding about the topic?	CBL	LBL					
5.	Which learning format kept you more engaged during the classes?	CBL	LBL					
E. Qualitative domain								
1.	How would you describe your understanding of pharmacological concepts when taught through CBL compared to LBL?							
2.	Can you reflect on how well you were able to recall and retain pharmacological information from both CBL and LBL?							
Follow-up: What strategies did you use to remember concepts taught in each method?”								
Follow-up: Was there any topic you could easily recall later because of how it was taught?”								
3.	In what ways did either teaching method encourage you to think critically or solve pharmacology-related problems?							
Follow-up: Situation where you used critical thinking during or after the classes to analyze a case study?								
Follow-up: Did you feel encouraged to ask questions								
4.	How did your engagement level vary between CBL and LBL?							
Follow-up: Which method kept your attention better during class?								
Follow-up: What made you feel more involved in a class? In what way								
5.	Can you share an example of a topic that felt more applicable or useful in real-life situations through either teaching method?							
Follow-up: Did discussing cases help you connect the topic to real-life scenarios?								
Follow-up: Did it motivate you to explore that topic further on your own?”								
6.	What aspects of each teaching method aligned best with your personal learning style, and why? Follow-up.							
Follow-up: What type of learning activities help you learn best: discussions, reading, listening, or applying concepts?								
Follow-up: Did you find either method more flexible or structured, and how did that affect you?								
Follow-up: Did you feel one method was easier to follow based on how you like to learn?”								
7.	Based on your experiences, what changes or improvements would you suggest for pharmacology teaching methods?							
Follow-up: Is there anything specific you would like to add to CBL or lectures to improve learning?								
Follow-up: Is there any challenge you faced that, if addressed, would improve your learning experience?								
8.	Which format, between CBL and traditional lectures, would you prefer not to use, and why?							
